# Optimization of the Green Extraction of Red Araçá (*Psidium catteyanum* Sabine) and Application in Alginate Membranes for Use as Dressings

**DOI:** 10.3390/molecules28186688

**Published:** 2023-09-18

**Authors:** Douglas Hardt Lacorte, Alaor Valério Filho, Márcio Dantas Carvalho, Luisa Bataglin Avila, Caroline Costa Moraes, Gabriela Silveira da Rosa

**Affiliations:** 1 Graduate Program in Science and Engineering of Materials, Federal University of Pampa, Bagé 96413-172, Brazil; douglaslacorte.aluno@unipampa.edu.br (D.H.L.); caroline.moraes@unipampa.edu.br (C.C.M.); 2 Graduate Program in Materials Science and Engineering, Technology Development Center, Federal University of Pelotas, Pelotas 96010-610, Brazil; alaorvf@msn.com; 3 Chemical Engineering, Federal University of Pampa, Bagé 96413-172, Brazil; marciocarvalho.aluno@unipampa.edu.br; 4 Department of Chemical Engineering, Federal University of Santa Maria, Santa Maria 97105-900, Brazil; luisabataglinavila@gmail.com

**Keywords:** bioactive compounds, biopolymer, skin lesion

## Abstract

In this research, the aim was to introduce innovation to the pharmaceutical field through the exploration of an underutilized plant matrix, the red araçá, along with the utilization of sodium alginate for the development of membranes designed for active topical dressings. Within this context, optimal extraction conditions were investigated using the central composite rotational statistical design (CCRD) to obtain a red araçá epicarp extract (RAEE) rich in bioactive compounds utilizing the maceration technique. The extract acquired under the optimized conditions (temperature of 66 °C and a hydroalcoholic solvent concentration of 32%) was incorporated into a sodium alginate matrix for the production of active membranes using a casting method. Characterization of the membranes revealed that the addition of the extract did not significantly alter its morphology. Furthermore, satisfactory results were observed regarding mechanical and barrier properties, as well as the controlled release of phenolic compounds in an environment simulating wound exudate. Based on these findings, the material produced from renewable matrices demonstrates the promising potential for application as a topical dressing within the pharmaceutical industry.

## 1. Introduction

The demand for natural products is on the rise, driven by their numerous health benefits and the economic allure of sustainable raw materials characterized by their abundance and potential for renewal. However, a significant portion of these sustainable matrices remains largely unexplored, and their true potential remains untapped. Consequently, there is a growing impetus for research aimed at obtaining natural products that not only contribute to reducing environmental impact but also seek to identify compounds with direct health benefits for humans while simultaneously lowering production costs.

Biopolymers are prime examples of materials sourced from renewable origins, encompassing microorganisms, algae, plants, and other natural sources. In the realm of alternative materials to synthetic polymers, alginates emerge as particularly noteworthy due to their inherent biocompatibility with active biocompounds. This biocompatibility enhances the antioxidant and antimicrobial properties of the resulting materials [1]. Alginic acid naturally occurs as the principal structural polysaccharide found in marine algae of the Phaeophyceae class. This polymer finds extensive application in various domains, including the food industry, dentistry, and biomedical fields. It is employed for purposes such as drug encapsulation and as a dressing material [2]. Sodium alginate membranes, when cross-linked with Ca^+2^, exhibit notable wound-healing capabilities attributed to their capacity for exudate absorption. Upon contact with the wound, an ionic exchange occurs between the calcium within the material and the sodium present in the exudate. This exchange facilitates blood circulation within undamaged blood vessels while also stemming bleeding through vasoconstriction, a process recognized as hemostasis. Moreover, the sustained moisture and hydration within the region further promote autolytic debridement. Consequently, the combined advantages of these factors, alongside the antioxidant and anti-inflammatory properties of plant matrices, endow this material with significant potential as a highly functional and sustainable solution [3].

Despite Brazil’s remarkable biodiversity, a substantial portion of both native and exotic species remains underexplored, with their true potential largely unknown [4,5]. Plants produce phytochemicals as a defense mechanism against predators, ultraviolet radiation, and environmental changes. These phytochemicals, or bioactive compounds, play a vital role in enhancing metabolic and physiological functions in the human body, making them valuable in various fields due to their antioxidant and anti-inflammatory properties [6,7]. The extraction of phytochemicals from plant matrices is typically accomplished through solid-liquid extraction. Efficient optimization of this process is crucial for ensuring the maximum yield of bioactive compounds [8]. Among the Brazilian native species with phenolic compounds in its composition is the araçá (*Psidium catteyanum* Sabine), a member of the *Myrtaceae* family. Red araçá stands out due to the higher anthocyanin content in its epicarp [9]. Among the phenolic acids present in the red araçá, there are gallic, ellagic, vanillic and p-coumaric acids, as well as the flavonoids quercetin, myrecithin, catechin and kaempferol, and anthocyanins cyanidin, malvidin and delphinidin, among others [10,11]. These compounds can act as active agents associated with biopolymeric materials for different applications.

As an example of research focused on this integration, Dias et al. [12] utilized commercial dressings composed of N-Carboxybutyl chitosan (CBC), collagen/cellulose (Promogran^®^, Saint Paul, MN, USA) and hyaluronic acid-based (Hyalofill^®^, Panama City, Panama) incorporated with jucá plant extract. The resulting material demonstrated anti-inflammatory activity. Karroubi et al. [13] prepared chitosan membranes with sodium alginate incorporated with *Teucrim polium* extract and obtained a healing rate of 98%, surpassing healing ointment and commercial elastic bandages. In terms of phytochemical release, Cirillo et al. [14] associated curcumin and sodium alginate and obtained satisfactory results, reinforcing the ability of alginate to release compounds of interest in a controlled environment.

The primary aim of this study is to rigorously investigate the extraction process by optimizing the phytochemical yield from red araçá extract. Furthermore, our research endeavors to pioneer the synthesis of sodium alginate membranes cross-linking and enrichment with red araçá extract, thus presenting an innovative avenue for potential application in advanced wound dressings.

## 2. Results and Discussion

### 2.1. Optimization of Red Araçá Epicarp Extraction

Table 1 presents the conditions for each run conducted, along with the Total Phenolic Content (TPC), Antioxidant Activity (AA), and Total Anthocyanin Content (TA) of the araçá extract. In comparison to previous similar studies, the araçá extract demonstrates highly promising results across all dependent variables. The TPC results from de araçá extract, between 18.44 and 31.06 mg_GAE_ g^−1^, were higher than those reported in the literature. For instance, Denardin et al. [8] achieved 6.6 mg GAE g^−1^ for the red araçá extract using ethanol 95% as a solvent in an orbital shaker. Also, Medina et al. [15] obtained 5.90 mg GAE g^−1^ for the red araçá fruit extract using a similar methodology but employing water as the solvent.

In the experimental design, TA values ranged from 6.21 to 41.97 mg_cn-3-glu_ 100 g^−1^. Meregalli et al. [16] reported a TA of 116 mg_cn-3-glu_ 100 g^−1^ by maceration of red araçá epicarp with ethanol 90% at 40 °C, which is higher than the result in the present study. This phenomenon can be attributed to the acidified solvent at pH 1.5, which fosters enhanced stability of anthocyanins. Nonetheless, extractions without chemical agents to alter pH are considered more eco-friendly and are also recommended for medical applications. The AA by the DPPH method presents values in the range of 82.04 to 95.10%RSC_DPPH_. In comparison, Medina et al. [15] and Meregalli et al. [16] reported AA values for araçá epicarp extract of 30.25 and 85.67%RSC_DPPH_, respectively. Both studies exhibited lower TPC, indicating that the presence of phenolics plays an important role in the AA of the extract. The AA value obtained by Meregalli et al. [16] was the most similar, which can be attributed to the high anthocyanin content extracted, underscoring the potent antioxidant action of this natural dye.

**Figure 1:**
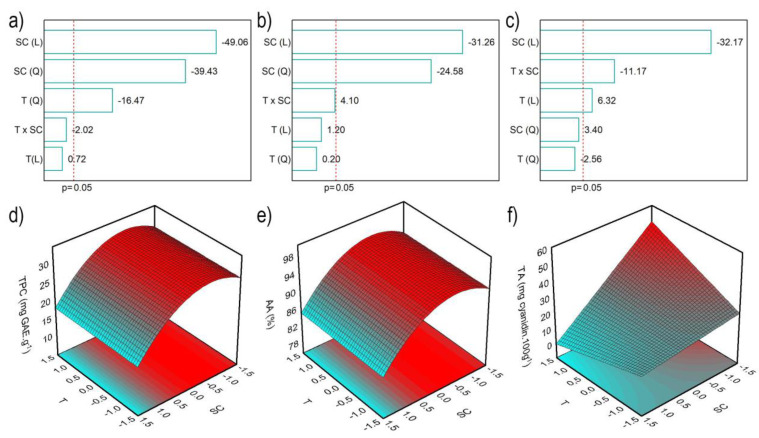
Pareto charts and the surface response of TPC (**a**,**d**), AA (**b**,**e**) and TA (**c**,**f**), respectively.

Pareto charts were employed to assess the effect of independent variables on the TPC, AA and TA (Figure 1a–c) with a significance of 95% (*p* < 0.05). In the TPC results, temperature (T) and solvent concentration (SC) showed significant negative effects. This result suggests that lower temperatures and ethanol concentration favor the TPC. For the AA and TA contents, the solvent concentration was the most influential effect.

The analysis of variance (ANOVA) of the second-order polynomial models was employed to confirm the statistical significance of the models under the selected operating conditions. All models demonstrated regression significance, as indicated by the F_ratio_ (F_value_/F_table_) higher than 1. Specifically, TPC, AA, and TA exhibited F-ratios of 15.81, 2.61, and 3.54, respectively.

Furthermore, the correlation coefficient (R²) obtained for the models are 0.967, 0.743 and 0.868 for TPC, AA and TA, respectively, i.e., the mathematical models generated for TPC, AA and TA were predictive. The respective mathematical expressions of these models are presented in Equations (1)–(3).

(1) $$TPC=30.92-1.43T^{2}-3.58SC-3.43{SC}^{2}$$

(2) $$AA=94.63-2.76SC-2.59{SC}^{2}$$

(3) TA=21.71+1.91T−9.73SC−4.77TSC

**Figure 2:**
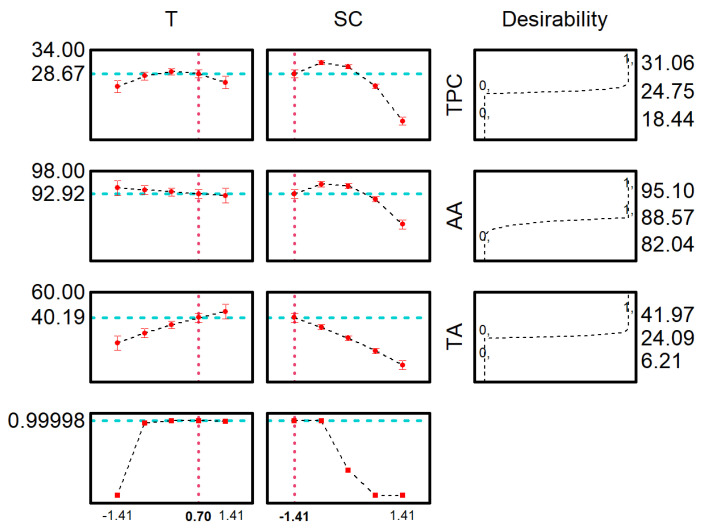
Global desirability function for the optimum conditions for TP, AA and TA.

Figure 2 shows that lower ethanol concentrations favor the extraction of phenolics and anthocyanins. These findings are in contrast with Meregalli et al. [16], whose research revealed that the increase in ethanol concentration, which varied between 35 and 90%, favored the extraction of phytochemicals from araçá epicarp at temperature of 4 °C. Castañeda-Valbuena et al. [17] explored ethanol concentrations from 35 to 90% in the ultrasonic extraction at room temperature of seeds and mango peel flour, observing better results at 80% ethanol concentration. When performing sinapine extraction from industrial mustard bran, Reungoat et al. [18] studied the temperature and concentration of ethanolic solvent. The authors conclude that the best condition was at a temperature of 75 °C and ethanol concentration of 70%, although they used a condensation column for extraction. Avila et al. [19] examined the effect of temperature and pH on the extraction of phytochemicals from jaboticaba peel using water as a solvent. In this case, the temperature was responsible for a positive increase in the final response, in agreement with the present work (TA). This behavior is related to the increase in temperature, which helps the extraction by diffusion [20].

The desirability function was employed to identify the optimal extraction conditions that simultaneously benefit all responses. The maximum global desirability (MGD) attained a value of 0.99, which is considered excellent according to the Harrington scale. The optimum conditions predicted by MGD (temperature of 66 °C and solvent concentration of 32%) were TPC of 28.67 mg_GAE_ g^−1^, AA of 92.93%RSC_DPPH_ and TA of 40 mg_cyanidin_ 100 g^−1^.

The red araçá epicarp extract obtained at optimized conditions was analyzed using FRAP and HPLC techniques. The antioxidant activity obtained by FRAP was 154.29 ± 8.99 µmol FeSO_4_ g^−1^ (d.b.). This result is in agreement with the values obtained in the literature. Using a previously described extraction method, Denardin et al. [8] estimated an AA of 89.09 µmol FeSO_4_ g^−1^. Valerio Filho et al. [21], through maceration of red araçá epicarp using 40% (*v*/*v*) ethanol as a solvent, 88 °C temperature, and a 2 h duration, obtained 222.53 ± 7.29 µmol FeSO_4_ g^−1^.

**Figure 3:**
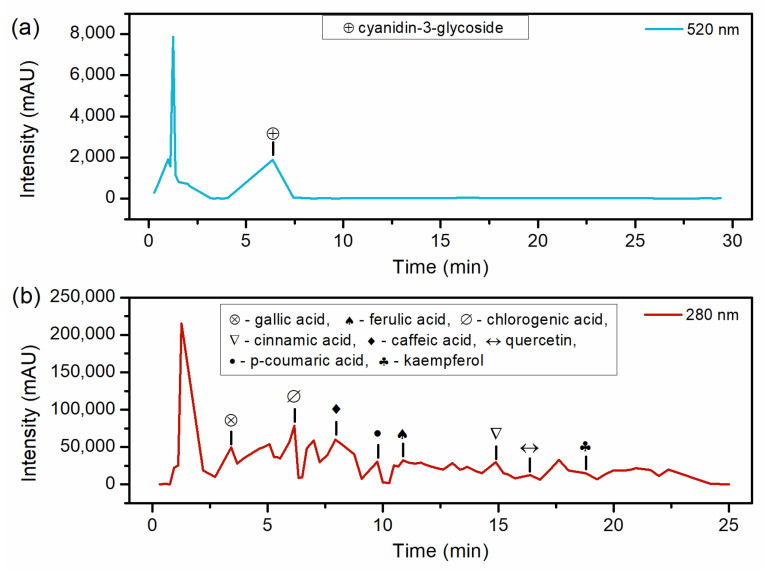
HPLC chromatograms of optimized araçá extract (**a**) at 520 nm indicating retention time of anthocyanins and (**b**) at 280 nm indicating retention time of phenolic acids and flavonoids.

Table 2 and Figure 3 present the HPLC results. Regarding the detected compounds, Cinnamic acid has not been previously reported in studies that evaluated red araçá extract. This finding is notable for the extraction method proposed in this work since Cinnamic acid had already been reported in *Myrtaceae* fruits, to which the red araçá belongs [22]. Concerning Gallic acid, the value of 0.136 mg g^−1^ obtained in this work is lower than the range reported in the literature. Ribeiro et al. [23] performed the extraction of the red araçá epicarp with absolute ethanol under magnetic stirring for 4 h at 25 °C and obtained 0.464 mg g^−1^, and Medina et al. [15] obtained 0.69 mg g^−1^ of gallic acid.

P-coumaric and Ferulic acids were not quantified in the present study because they did not reach the minimum detection limit. Ribeiro et al. [23] quantified 0.115 mg g^−1^ of quercetin, lower than in the present study. Quercetin is a compound of interest in various studies due to its anti-inflammatory and antioxidant properties [24,25]. Regarding kaempferol, Pereira et al. [10] identified 0.0002 mg g^−1^ in yellow araçá using extraction methanol for 5 min under vortexing. Anthocyanins are responsible for the coloration of several fruits [19,26], and Cyanidin-3-glucoside is the most abundant anthocyanin in the red araçá epicarp. Dalla Nora et al. [27] quantified 0.354 mg g^−1^ in the extract of red araçá fruit using 1% concentrated hydrochloric acid in methanol at room temperature, a lower value than that shown in Table 2. The variation in the composition of extracted compounds can be attributed to several influencing factors. Araçá cultivation conditions, such as soil composition, climate, and agricultural practices, can significantly impact the phytochemical profile of the fruit. Additionally, the degree of fruit maturation and variations in the extraction technique, including the choice of solvent, extraction temperature, and duration, can lead to differences in the final composition of the extract. Lastly, differences in HPLC operating conditions, such as column type and mobile phase composition, can influence the detection and quantification of specific compounds.

The literature contains reports attributing antioxidant activity to various compounds, including Gallic acid [28], Chlorogenic acid [29], caffeic acid [30], Ferulic acid [21], Cinnamic acid [31], Quercetin and Kaempferol [32], and Cyanidin-3-glucoside [33]. The antioxidant action attributed to phenolic compounds occurs by inactivating free radicals through the donation of hydrogen atoms. Free radicals are responsible for several degenerative diseases. Consequently, the extract of red araçá epicarp exhibits potential for use as an antioxidant additive in the formulation of products for the food, medical, and therapeutic industries, owing to the presence of numerous compounds that have been extensively studied in the literature for their antioxidant properties.

### 2.2. Membrane Characterization

Considering the properties of the optimized extract, sodium alginate membranes were synthesized in two formulations: one containing araçá extract (ME) and the other without (MC). The results of this evaluation are shown in Table 3.

The thickness of membranes did not exhibit a significant difference by the Tukey test. This behavior is also reported by Kharroubi et al. [13], who developed gentamicin-loaded arabinoxylan-sodium alginate films for wound dressing using different formulations. The values obtained from ME and MC are close to the 0.108 mm for sodium alginate membrane obtained by the casting method performed by Pacheco et al. [34]. Marangoni Junior et al. [35] also reported similar thickness values of 0.131 and 0.144 mm for the control film of sodium alginate and films of sodium alginate with green propolis extract, respectively. Moreover, considering the average thickness of human skin ranges from 0.2 to 0.5 mm, membranes with thicknesses, as described in Table 3, can be promising to use as skin dressing since the thin films are more comfortable for patients [13,36].

WVP results were 2.479 × 10^−9^ and 1.906 × 10^−9^ g/m.s.Pa, for MC and ME membranes, respectively. The literature reports similar values for sodium alginate membranes cross-linked with Ca^+^. Rhim [37] developed sodium alginate membranes cross-linked with 1% (*w*/*v*) CaCl_2_ solution, reaching a value of 1.0870 × 10^−9^ g/m.s.Pa. The cross-linking step promotes a positive impact on the membrane barrier, being capable of reducing polymer segmental mobility and, consequently, WVP [37]. Mahcene et al. [38] developed a sodium alginate membrane and reported a value of 1.667 × 10^−12^ g/m.s.Pa and from alginate membranes with different essential oils, values ranging from 1.111 × 10^−12^ to 2.5 × 10^−12^ g/m.s.Pa. The differences between these values and those obtained in the present study could be attributed to variations in the concentration of the polymeric solution used in the manufacturing process.

WVP values are significantly different, demonstrating a reduction in this parameter by the addition of red araçá extract. This result was expected since the polyphenols present in the extract, as observed in their characterization, promote interaction with the biopolymer. This interaction possibly occurs through hydrogen or covalent bondings, consequently limiting the availability of hydrogen groups and reducing the affinity of the biopolymeric membrane with water. Therefore, lower values in mass diffusion result in a reduction in water vapor permeability [19,39].

The WVPR follows the same behavior as the WVP. These results are higher than those described by the literature. Kaczmarek [40] developed sodium alginate membranes with and without tannic acid and found values ranged from 0.0045 to 0.0067 g/m².s. Pacheco et al. [34] also developed sodium alginate membranes incorporating diclofenac sodium (DS) with values of 0.0136 and 0.0272 g/m².s for sodium alginate membranes without DS and with DS, respectively. The results obtained in the present study suggest that the developed membranes are permeable to water vapor and capable of guaranteeing a humid environment at the wound site, avoiding excessive dehydration. Thus, MC and ME correspond to the desired requirement for a dressing that enhances the wound healing process through its ability to maintain an optimal moisture environment. According to Lamke, Nilsson and Reithner [41], the loss of water by evaporation of normal skin is approximately 0.0024 g/m².s, while injured skin can reach a rate of water loss of up to 0.0595 g/m².s, depending on the degree of injury. Furthermore, the optimal WVTR range of a wound dressing, considering effective wound healing, has been reported to be 0.0231–0.0289 g/m².s [36,42,43]. So, it is possible to observe that the values obtained are close to the ideal range of WVTP, and the addition of natural extract contributed to improving this parameter.

The water solubility of membranes exhibited values of 50.18 and 41.43 for MC and ME, respectively. The result for the MC sodium alginate membrane aligns with the reported value of 49.7 ± 0.2% by Chen et al. [44]. These authors also described the same behavior in sodium alginate membranes added with thymol. In contrast, Cheng et al. [45] reported 99.61 ± 1.48% for sodium alginate membranes, and values ranged from 96.82 ± 1.72 to 90.43 ± 1.33% for sodium alginate membranes with different concentrations of β-cyclodextrin/carvacrol microcapsules. Thus, the results presented in Table 3 are favorable for application as dressings, as skin wounds are known to release exudate, with which the material will come into contact.

The membranes were also evaluated for their mechanical properties, including tensile strength and elongation at the breakpoint. The values for tensile strength were 8.468 and 6.676 MPa for the control membrane and membrane with the extract. These results surpass those reported by Bakar et al. [46], who reported a value of 4.0 MPa for sodium alginate membrane and ranged from 4.0 to 5.1 MPa for sodium alginate membranes with different concentrations of Ageratum conyzoides extract. Rezvanian et al. [47] also evaluated the mechanical properties of sodium alginate membranes with and without simvastatin as well as composite membranes (sodium alginate/pectin and sodium alginate/gelatin) with and without simvastatin and reported values ranged from 0.89 ± 0.05 to 3.32 ± 0.58 MPa. In the present study, it is possible to observe the non-significant reduction in tensile strength due to the addition of natural extract. Avila et al. [48] observed the same behavior for carrageenan films additive with jaboticaba peel extract at different concentrations. According to Chi et al. [49], this phenomenon may be attributed to the molecules present in the red araçá extract that interact with the polymeric matrix. Hassan et al. [50] and Abbasi et al. [51] suggested that the tensile strength of normal skin is around 11.5 MPa.

Regarding the elongation at break, the values observed in the present study were 15.372 ± 2.575% for MC and 8.190 ± 1.468% for ME. These findings are consistent with those reported by Rhim et al. [37], who developed cross-linked membranes by two methods (mixing film and immersion film) and obtained values ranging from 8.2 ± 2.2% to 3.4 ± 1.4% for cross-linked sodium alginate membranes in different concentrations of CaCl_2_ solutions. In contrast, Pacheco et al. [34] reported lower values than observed in the present study. The authors found values of 2.76 ± 1.33% for the control membrane and 1.94 ± 0.27% for the membrane incorporated with diclofenac sodium. Besides that, it is possible to observe a significant decrease in this parameter by the addition of red araçá extract.

**Figure 4:**
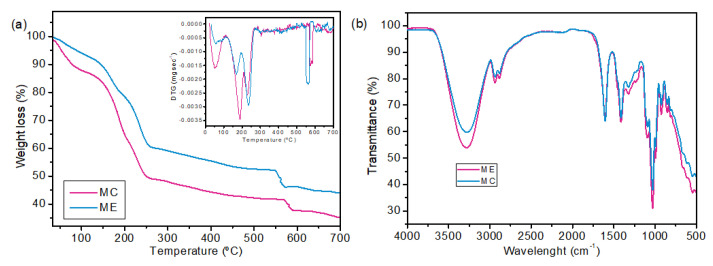
Characterizations of MC and ME membranes. Thermogravimetric curves (**a**), FTIR spectrum (**b**).

The thermogravimetric analysis is shown in Figure 4a, and the FTIR spectrum is presented in Figure 4b.

The TG and DTG curves indicate that the membrane with araçá extract showed greater thermal resistance than the control membrane, with 65% and 56% degradation for MC and ME, respectively. The first peak of mass loss occurred between 25 and 110 °C for both samples, indicating mass loss of moisture and volatile compounds. However, MC showed higher mass loss at this stage. This result can be due to the activities of reduced and intermediate water occurring between the active sorption sites, which, in the case of alginate, are the COO-Na+ and -OH functional groups [52]. Part of these functional groups probably interacts with the phenolic compounds of the araçá extract. In this case, the ME sample has less interaction with water and, consequently, less moisture retention. The third weight loss at 225–235 °C corresponds to a complex process, which led to the degradation of the glycoside bond, resulting in the formation of H_2_O, CH_4_ and CO_2_ [53].

FTIR analysis was applied in this study to identify functional groups present on the sample surfaces. Despite the similarity between the spectra, the main differences can be seen at 3600–3000 cm^−1^ and 600–500 cm^−1^, which indicate the presence of O-H and N-H stretching and the presence of polyphenols of araçá extract, respectively [21,54]. The bands at 2937 cm^−1^ and 2883 cm^−1^ correspond to the asymmetrical and symmetrical stretching vibrations of the CH_3_ and CH_2_ groups. The characteristic bands of alginate were identified. The asymmetric and symmetric stretching vibration of COO- at 1605 cm^−1^ and the C-O of the glucopyronose ring at 1411 cm^−1^ [53].

**Figure 5:**
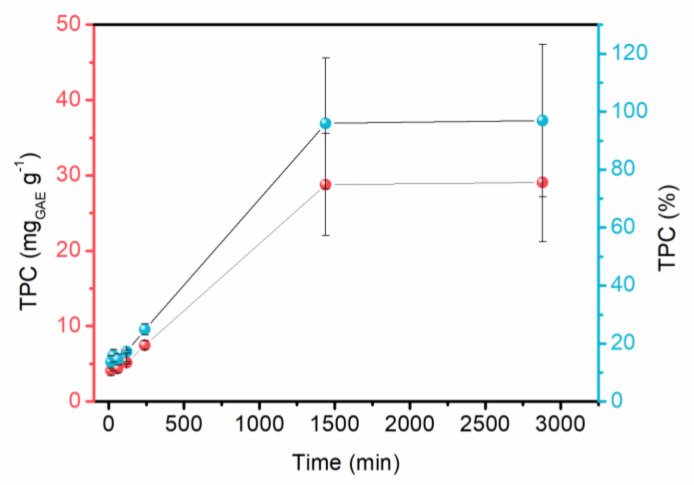
Release of bioactive compounds from the membrane (ME).

In the phytochemicals release analysis (Figure 5), a slow and constant release was observed, reaching its maximum at 24 h. The results are promising for the desired application, as topical dressings are usually changed daily. The araçá extract has the presence of several antioxidant and anti-inflammatory compounds, especially quercetin. These released compounds can act to benefit wound healing. The analysis was performed with the membrane immersed in simulated exudate at 37 °C (human body temperature). However, it is well known that there will be less exudate in real applications, with an ionic exchange between sodium in the wound and calcium in the cross-linked membrane. This allows the released exudate to be absorbed by the alginate due to its gelling capacity. In this exchange, phytochemicals are expected to be released into the wound.

Some works have explored the release of drugs and phytochemicals from materials produced with sodium alginate using phosphate-buffered saline (PBS) solution under constant agitation at 37 °C. Alzarea et al. [55] investigated the release of the antibiotic gentamicin from alginate films and observed 40% drug release in the first hour, with maximum release in 24 h. Dong et al. [56] incorporated ciprofloxacin hydrochloride in alginate membranes and evaluated parameters that affect release, with medium pH, film thickness, amount of incorporated drug and cross-linking time being relevant factors. These factors, if optimized, can significantly improve the release of compounds into the wound. The limited studies available in the literature for cross-linked alginate membranes suggested that the polymer has a good capacity to release drugs and bioactive compounds, being promising for use in dressings and as a coating for drug capsules.

**Figure 6:**
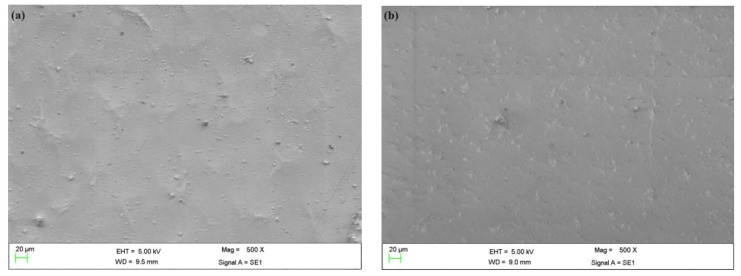
SEM of membranes, MC (**a**), ME (**b**).

Figure 6 shows scanning electron microscopy for MC and ME at 500× magnification.

In the SEM images, it is evident that the extract did not induce differences in the material’s surface. The apparent roughness on the surface could be related to the cross-linking process [57]. A crucial characteristic for the release of compounds in dressings is porosity. While the material itself may not exhibit porosity, alginate is highly hydrophilic, and following cross-linking and upon contact with the wound, it has the capability to form a gel, thereby facilitating the release of the enclosed compounds. Pacheco et al. [34] observed a material with a uniform surface for cross-linked sodium alginate membranes without additives and membranes with greater roughness with the additive sodium diclofenac.

Based on the study’s findings, several future research directions are evident. These include a deeper exploration of the mechanisms controlling the release of bioactive compounds from sodium alginate-based materials, especially in conditions simulating real wound environments. Furthermore, assessing the long-term stability and effectiveness of sodium alginate membranes as wound dressings in both preclinical and clinical contexts is crucial for practical applicability.

## 3. Materials and Methods

### 3.1. Materials

The red araçá fruits were harvested at coordinates −31.553710°, −53.683664°, in the city of Candiota, Rio Grande do Sul, Brazil, in April 2021. Sodium Alginate with viscosity between 300–400 CPs at 20 °C was obtained from Êxodo Científica. Ethanol, glycerol, 2,2-diphenyl-1-picrylhydrazyl (DPPH), Folin-Ciocalteau, anhydrous sodium carbonate, calcium chloride, anhydrous ferric chloride, sodium acetate, hydrochloric acid, acetic acid, 2,4,6-tripiridil-s-triazina (TPTZ) were obtained from Sigma Aldrich (St. Louis, MO, USA) with analytical grade. Ultra-pure water, methanol, acetonitrile, gallic acid, cyanidin-3-glucoside, chlorogenic acid, caffeic acid, p-coumaric acid, ferulic acid, cinnamic acid, quercetin and kaempferol were purchased from Sigma Aldrich (St. Louis, MO, USA) with HPLC grade.

### 3.2. Extraction Procedure

Initially, the red araçá fruits were sanitized with 2% sodium hypochlorite, followed by washing with distilled water. The epicarp of the fruits was manually separated and freeze-dried for 24 h at −50 °C (Terroni, LS3000, São Carlos, Brazil). Subsequently, particles were reduced in an analytical mill (IKA, A11, Darmstadt, Germany) and sieved (60 mesh, Metallurgical Industry Bertel, Caieiras, Brazil). The particles with a diameter smaller than 0.250 mm were used in the extraction. The maceration extraction methodology was adopted using a metabolic bath (Dubnoff, QUIMIS, Diadema, Brazil) with constant stirring and heating. The extraction was carried out with 50 mL of hydroalcoholic solvent (without changing the pH) and 1 g of araçá epicarp for 2 h. Then, the extracts were vacuum filtered with Whatman filter paper (Fisher Scientific, Hampton, VA, USA).

The extraction was evaluated through a central composite rotational design (CCRD) by STATISTICA 13.5 software (SAS Institute, Cary, NC, USA), varying temperature (T) and solvent concentration (SC) with a range of 34 to 76 °C and 32 to 92% ethanol *v*/*v*, respectively. The dependent variables, TPC, AA and TA, were characterized in triplicate. The adequacy of the models was analyzed by variance analysis (ANOVA) and the Fisher test. The simultaneous optimization of all dependent variables was performed by the global desirability methodology (GDM).

### 3.3. Extracts Characterization

Total phenolic compounds quantification was performed using Folin-Ciocalteau [58], and the results were estimated with a gallic acid standard curve (50 to 1000 mg/mL) and expressed in mg_GAE_ g^−1^. The extract was subjected to the DPPH free radical reduction method to determine antioxidant activity [59], and the result was expressed as a percentage of DPPH radical scavenging capacity (% RSCDPPH). Total anthocyanin content was assessed by absorbance measurements of the extract in a spectrophotometer at a wavelength of 520 nm. The results were estimated using the cyanidin-3-glucoside standard curve (0.005 to 0.1 mg/mL), expressed as mg of cyanidin-3-glucoside per 100 g sample.

The extract obtained under optimized conditions was also characterized by Ferric Reducing Antioxidant Power (FRAP) and High-Performance Liquid Chromatography (HPLC). FRAP solution was formulated using 0.3 M acetate buffer, TPTZ and 20 mM ferric chloride. For the analysis, three different dilutions were made in the extracts. FRAP reagent was prepared at the time of the reaction with a 10:1:1 ratio of acetate buffer, TPTZ and ferric chloride. 100 µL of each extract dilution, 300 µL of distilled water and 3 mL of FRAP reagent were added to test tubes. The mixtures were sent to the water bath (Q215M2, QUIMIS, Diadema, Brazil) at 37 °C for 30 min and analyzed in a spectrophotometer at a wavelength of 595 nm. The results obtained were analyzed using the standard curve of ferrous sulfate and expressed in µmol of ferrous sulfate per g of sample (µM Fe^+2^ g^−1^).

HPLC measurements were performed using centrifuged extracts (Q222TM2, QUIMIS, Diadema, Brazil). Then, the extracts were filtered with a 0.45 µm syringe filter and transferred to specific vials for chromatography. The equipment used (Agilent 1260 Series, Santa Clara, EUA) is equipped with a quaternary pump and a diode array detector (DAD). Separation took place on an Eclipse Plus C18 (Supelco, Bellefonte, PA, USA) reversed-phase LC column. The temperature was 30 °C, with an eluent flow rate of 1 mL.min^−1^, and the injected sample volume was 20 µL. The solvents used for the separation were 0.2% acetic acid (A), methanol P.A. (B) and acetonitrile P.A. (C). The detection of phytochemicals was performed at wavelengths of 280 and 520 nm for phenolics and anthocyanins, respectively. Phytochemicals were quantified using calibration curves with chromatographic standards, as follows: gallic acid, chlorogenic acid, caffeic acid, p-coumaric acid, ferulic acid, cinnamic acid, quercetin, kaempferol and cyanidin-3-glucoside.

### 3.4. Preparation and Characterization of Sodium Alginate Membranes

Initially, 5 g of sodium alginate was solubilized and homogenized in 200 mL of distilled water using a mechanical stirrer at 600 rpm for 1 h [34]. During this homogenization, 5 g of glycerol and 1 g of freeze-dried optimized extract were added. Then, the membrane solution was completed up to 250 mL using distilled water and set to rest for 30 min. After this stage, the solution was forwarded to rectangular acrylic plates measuring 23 × 23 cm and dried in a forced convection oven at 40 °C for 24 h. After drying, the membranes were cross-linked to decrease their solubility in water using a solution of 0.1% CaCl_2_ and 1% glycerol for 1 h. Finally, they were placed in a forced convection oven at 40 °C for 12 h. The membranes synthesized with araçá extract (ME) and the control membrane (MC) were properly stored in a desiccator at 25 °C and a controlled humidity of 50% until being characterized.

The thickness of the membranes was obtained with a digital micrometer (Insize, model IP65), with ten measurements. The water vapor permeability (WVP) test was performed in the laboratory following the E96-96 standards [60]. The membranes were attached to the top of the flasks using 30 g of anhydrous calcium chloride (CaCl_2_). The flasks were placed in desiccators, where the relative humidity of the medium was maintained at 50%. After samples remained for 10 days in a desiccator, they were weighed to verify the CaCl_2_ mass gain. The water solubility of the membranes was determined using the proposed method of Gontard et al. [61] with modifications. Initially, the 2 × 2 cm membrane samples were dried in a forced circulation oven at 60 °C for 24 h and weighed. Afterward, the samples were immersed in a container with 50 mL of distilled water under agitation at 50 rpm for 24 h at room temperature. The suspension was filtered, and the residue dried at a temperature of 60 °C for 24 h and weighed. A tensile test was carried out following the D882 standard [62] with a texturometer (Stable Micro Systems, model TA.TX, Godalming, UK). The TGA was performed in a thermogravimetric analyzer (Shimadzu brand, model TGA-50, Kyoto, Japan) to determine the mass loss as a function of temperature. The analysis was carried out in an inert nitrogen atmosphere at a flow rate of 60 mL min^−1^ in the range of 25 to 700 °C, at a heating rate of 10 °C min^−1^ [63]. Fourier Transform Infrared Spectroscopy (FTIR, Prestige 21, Nakagyo-ku, Kyoto, Japan) was used to identify the functional groups on the sodium alginate membranes. The spectrum was performed in the range of 500–4000 cm^−1^ with a resolution of 4 cm^−1^.

To verify the release of phytochemicals in the membrane, a solution was used to simulate the exudate of skin wounds (142 mmol.L of Na and 2.5 mmol.L of Ca), using NaCl and CaCl_2_ for its preparation. The membranes were cut into 2 × 2 cm and immersed in the solution, remaining under constant stirring at a temperature of 37 °C for times between 15 min and 24 h. Afterward, aliquots were removed, and the TPC was analyzed to verify the release of phytochemicals in the medium.

## 4. Conclusions

The optimization of the green extraction process for obtaining bioactive compounds has proved effective and showed that the solvent concentration was the most influential factor. Notably, the results highlighted that lower concentrations of ethanol improved the process. The mathematical models derived from the experimental design were predictive and capable of optimizing red araçá epicarp’s phytochemicals extraction. High-Performance Liquid Chromatography (HPLC) analysis revealed the presence of quercetin and antioxidant phenolic acids in the extract.

Sodium alginate membranes cross-linked exhibited good mechanical resistance, malleability and favorable thickness for use as dressings. It was possible to decrease the water solubility of the material from 50 to 41% for MC and ME. Moreover, the material was found to be permeable to water vapor, contributing to a humid environment at the wound site. In addition, results of the phytochemicals release test showed a slow and constant release after 24 h (approximately 29 mg_GAE_.g^−1^). These findings not only validate the viability of the green extraction process and sodium alginate membranes but also offer promising results for future advanced dressings.

## Figures and Tables

**Table 1:** DCCR with coded factors and real values with their respective TPC, AA and TA responses for red araçá extract.

	Coded Values		Real Values				
Run	T	SC	T (°C)	SC (%)	TPC (mg_GAE_ g^−1^, d.b)	AA (% RSC_DPPH_)	TA (mg_cn-3-glu_ 100 g^−1^, d.b)
1	−1	−1	40	−40	29.94 ± 1.21	94.25 ± 0.45	25.00 ± 0.15
2	1	−1	70	−40	31.06 ± 0.87	93.39 ± 0.27	41.97 ± 0.96
3	−1	1	40	84	22.99 ± 0.61	91.42 ± 0.65	8.34 ± 0.06
4	1	1	70	84	23.27 ± 0.82	92.61 ± 1.12	6.21 ± 0.06
5	0	0	55	62	30.69 ± 0.89	94.33 ± 0.35	22.28 ± 0.70
6	0	0	55	62	31.06 ± 0.60	94.80 ± 0.11	23.79 ± 0.50
7	0	0	55	62	31.02 ± 0.03	31.02 ± 0.03	94.71 ± 0.08
8	−1.41	0	34	62	27.65 ± 0.12	93.58 ± 0.23	22.02 ± 2.18
9	1.41	0	76	62	26.96 ± 1.27	93.95 ± 0.30	22.32 ± 0.43
10	0	−1.41	55	32	28.22 ± 2.54	95.10 ± 0.35	30.52 ± 0.10
11	0	1.41	55	92	18.44 ± 0.46	82.04 ± 1.43	12.61 ± 0.04

Data reported are the average of three replicates and ± mean deviation.

**Table 2:** Phenolic compounds identified and quantified by HPLC.

Compounds	Retention Time (min)	Concentration (mg/g d.b.)
Cyanidin-3-glucoside	6.37	0.904 ± 0.02
Gallic acid	2.7	0.136 ± 0.01
Chlorogenic acid	6.34 and 6.48	0.168 ± 0.004
Caffeic acid	7.26	0.118 ± 0.001
P-coumaric acid	9.08	ND
Ferulic acid	9.98 and 10.26	ND
Cinnamic acid	15.73	0.034 ± 0.001
Quercetin	16.38 and 16.8	0.657 ± 0.002
Kaempferol	19.28	0.355 ± 0.03

ND—below the limit of quantification.

**Table 3:** Properties of sodium alginate membranes with extract (ME) and without (MC).

	MC	ME
Thickness (mm)	0.135 ± 0.022 ^a^	0.1172 ± 0.016 ^a^
WVP (g/m.s.Pa)	2.479 × 10^−9^ ± 5.940 × 10^−11 a^	1.906 × 10^−9^ ± 2.244 × 10^−10 b^
WVPR (g/m².s)	0.0642 ± 0.0015 ^a^	0.0568 ± 0.0066 ^a^
Water solubility (%)	50.18 ± 1.86 ^a^	41.43 ± 0.67 ^b^
Tensile strength (MPa)	8.468 ± 1.248 ^a^	6.676 ± 0.360 ^a^
Elongation at break (%)	15.372 ± 2.575 ^a^	8.190 ± 1.468 ^b^

Mean ± mean deviation (*n* = 2). Different letters in the same column indicate significant differences between the samples for the *t*-test (*p* < 0.05).

## Data Availability

Not applicable.

## References

[1] Kamoun E.A., Kenawy E.R.S., Chen X. (2017). A Review on Polymeric Hydrogel Membranes for Wound Dressing Applications: PVA-Based Hydrogel Dressings. J. Adv. Res..

[2] Şen F., Uzunsoy İ., Baştürk E., Kahraman M.V. (2017). Antimicrobial Agent-Free Hybrid Cationic Starch/Sodium Alginate Polyelectrolyte Films for Food Packaging Materials. Carbohydr. Polym..

[3] Saraiva M.M., Campelo M.d.S., Câmara Neto J.F., Lima A.B.N., Silva G.d.A., Dias A.T.d.F.F., Ricardo N.M.P.S., Kaplan D.L., Ribeiro M.E.N.P. (2023). Alginate/Polyvinyl Alcohol Films for Wound Healing: Advantages and Challenges. J. Biomed. Mater. Res. B Appl. Biomater..

[4] Valli M., Russo H.M., Bolzani V.d.S. (2018). The Potential Contribution of the Natural Products from Brazilian Biodiversity to Bioeconomy. An. Acad. Bras. Cienc..

[5] Schulz M., Seraglio S.K.T., Brugnerotto P., Gonzaga L.V., Costa A.C.O., Fett R. (2020). Composition and Potential Health Effects of Dark-Colored Underutilized Brazilian Fruits—A Review. Food Res. Int..

[6] Raks V., Al-Suod H., Buszewski B. (2018). Isolation, Separation, and Preconcentration of Biologically Active Compounds from Plant Matrices by Extraction Techniques. Chromatographia.

[7] Das A.B., Goud V.V., Das C. (2017). Extraction of Phenolic Compounds and Anthocyanin from Black and Purple Rice Bran (*Oryza Sativa* L.) Using Ultrasound: A Comparative Analysis and Phytochemical Profiling. Ind. Crops Prod..

[8] Denardin C.C., Hirsch G.E., Da Rocha R.F., Vizzotto M., Henriques A.T., Moreira J.C.F., Guma F.T.C.R., Emanuelli T. (2015). Antioxidant Capacity and Bioactive Compounds of Four Brazilian Native Fruits. J. Food Drug Anal..

[9] Scur M.C., Pinto F.G.S., Pandini J.A., Costa W.F., Leite C.W., Temponi L.G. (2016). Atividade Antimicrobiana e Antioxidante Do Óleo Essencial e Diferentes Extratos Vegetais de *Psidium cattleianum* Sabine. Braz. J. Biol..

[10] Pereira E.d.S., Vinholes J.R., Camargo T.M., Nora F.R., Crizel R.L., Chaves F., Nora L., Vizzotto M. (2020). Characterization of Araçá Fruits (*Psidium cattleianum* Sabine): Phenolic Composition, Antioxidant Activity and Inhibition of α-Amylase and α-Glucosidase. Food Biosci..

[11] Mallmann L.P., Tischer B., Vizzotto M., Rodrigues E., Manfroi V. (2020). Comprehensive Identification and Quantification of Unexploited Phenolic Compounds from Red and Yellow Araçá (*Psidium cattleianum* Sabine) by LC-DAD-ESI-MS/MS. Food Res. Int..

[12] Dias A.M.A., Rey-Rico A., Oliveira R.A., Marceneiro S., Alvarez-Lorenzo C., Concheiro A., Júnior R.N.C., Braga M.E.M., De Sousa H.C. (2013). Wound Dressings Loaded with an Anti-Inflammatory Jucá (*Libidibia ferrea*) Extract Using Supercritical Carbon Dioxide Technology. J. Supercrit. Fluids.

[13] Kharroubi M., Bellali F., Karrat A., Bouchdoug M., Jaouad A. (2021). Preparation of Teucrium Polium Extract-Loaded Chitosan-Sodium Lauryl Sulfate Beads and Chitosan-Alginate Films for Wound Dressing Application. AIMS Public Health.

[14] Cirillo G., Curcio M., Oliviero Rossi C., De Filpo G., Baratta M., De Luca M., Iemma F., Nicoletta F.P. (2023). Curcumin–Sodium Alginate and Curcumin–Chitosan Conjugates as Drug Delivery Systems: An Interesting Rheological Behaviour. Molecules.

[15] Medina A.L., Haas L.I.R., Chaves F.C., Salvador M., Zambiazi R.C., Da Silva W.P., Nora L., Rombaldi C.V. (2011). Araçá (*Psidium cattleianum* Sabine) Fruit Extracts with Antioxidant and Antimicrobial Activities and Antiproliferative Effect on Human Cancer Cells. Food Chem..

[16] Meregalli M.M., Puton B.M.S., Camera F.D.M., Amaral A.U., Zeni J., Cansian R.L., Mignoni M.L., Backes G.T. (2020). Conventional and Ultrasound-Assisted Methods for Extraction of Bioactive Compounds from Red Araçá Peel (*Psidium cattleianum* Sabine). Arab. J. Chem..

[17] Castañeda-Valbuena D., Ayora-Talavera T., Luján-Hidalgo C., Álvarez-Gutiérrez P., Martínez-Galero N., Meza-Gordillo R. (2021). Ultrasound Extraction Conditions Effect on Antioxidant Capacity of Mango By-Product Extracts. Food Bioprod. Process..

[18] Reungoat V., Gaudin M., Flourat A.L., Isidore E., Mouterde L.M.M., Allais F., Ducatel H., Ioannou I. (2020). Optimization of an Ethanol/Water-Based Sinapine Extraction from Mustard Bran Using Response Surface Methodology. Food Bioprod. Process..

[19] Avila L.B., Fontes M.R.V., Zavareze E.d.R., Moraes C.C., Morais M.M., da Rosa G.S. (2020). Recovery of Bioactive Compounds from Jaboticaba Peels and Application into Zein Ultrafine Fibers Produced by Electrospinning. Polymers.

[20] Favaro G., De Leo D., Pastore P., Magno F., Ballardin A. (2008). Quantitative Determination of Chlorophenols in Leather by Pressurized Liquid Extraction and Liquid Chromatography with Diode-Array Detection. J. Chromatogr. A.

[21] Filho A.V., Avila L.B., Lacorte D.H., Martiny T.R., Rosseto V., Moraes C.C., Dotto G.L., Carreno N.L.V., da Rosa G.S. (2022). Brazilian Agroindustrial Wastes as a Potential Resource of Bioative Compounds and Their Antimicrobial and Antioxidant Activities. Molecules.

[22] Tuncel N.B., Yılmaz N. (2015). Optimizing the Extraction of Phenolics and Antioxidants from Feijoa (*Feijoa sellowiana*, Myrtaceae). J. Food Sci. Technol..

[23] Ribeiro A.B., Chisté R.C., Freitas M., Da Silva A.F., Visentainer J.V., Fernandes E. (2014). *Psidium cattleianum* Fruit Extracts Are Efficient in vitro Scavengers of Physiologically Relevant Reactive Oxygen and Nitrogen Species. Food Chem..

[24] Lesjak M., Beara I., Simin N., Pintać D., Majkić T., Bekvalac K., Orčić D., Mimica-Dukić N. (2018). Antioxidant and Anti-Inflammatory Activities of Quercetin and Its Derivatives. J. Funct. Foods.

[25] Yong-Bing X., Gui-Lin C., Ming-Quan G. (2019). Antioxidant and Anti-Inflammatory Activities of the Crude Extracts of Moringa Oleifera from Kenya and Their Correlations with Flavonoids. Antioxidants.

[26] Rodrigues L.M., Romanini E.B., Silva E., Pilau E.J., da Costa S.C., Madrona G.S. (2020). *Camu-Camu* Bioactive Compounds Extraction by Ecofriendly Sequential Processes (Ultrasound Assisted Extraction and Reverse Osmosis). Ultrason. Sonochem.

[27] Nora C.D., Müller C.D.R., de Bona G.S., Rios A.d.O., Hertz P.F., Jablonski A., De Jong E.V., Flôres S.H. (2014). Effect of Processing on the Stability of Bioactive Compounds from Red Guava (*Psidium cattleyanum* Sabine) and Guabiju (*Myrcianthes pungens*). J. Food Compos. Anal..

[28] Fernandes-Negreiros M.M., Batista L.A.N.C., Viana R.L.S., Sabry D.A., Paiva A.A.O., Paiva W.S., Machado R.I.A., de Sousa Junior F.L., Pontes D.d.L., Vitoriano J.d.O. (2020). Gallic Acid-Laminarin Conjugate Is a Better Antioxidant than Sulfated or Carboxylated Laminarin. Antioxidants.

[29] Qie X., Chen W., Zeng M., Wang Z., Chen J., Goff H.D., He Z. (2021). Interaction between β-Lactoglobulin and Chlorogenic Acid and Its Effect on Antioxidant Activity and Thermal Stability. Food Hydrocoll..

[30] Otan Özden F., Lütfioğlu M., Demir E., Bilgici B. (2021). Antioxidant Effect of Caffeic Acid Phenethyl Ester in Experimentally Induced Periodontitis. Clin. Oral. Investig..

[31] Babaeenezhad E., Nouryazdan N., Nasri M., Ahmadvand H., Moradi Sarabi M. (2021). Cinnamic Acid Ameliorate Gentamicin-Induced Liver Dysfunctions and Nephrotoxicity in Rats through Induction of Antioxidant Activities. Heliyon.

[32] Tian C., Liu X., Chang Y., Wang R., Lv T., Cui C., Liu M. (2021). Investigation of the Anti-Inflammatory and Antioxidant Activities of Luteolin, Kaempferol, Apigenin and Quercetin. S. Afr. J. Bot..

[33] Jaiswal V., Park M., Lee H.J. (2021). Comparative Transcriptome Analysis of the Expression of Antioxidant and Immunity Genes in the Spleen of a Cyanidin 3-o-Glucoside-Treated Alzheimer’s Mouse Model. Antioxidants.

[34] Pacheco M.S., da Silva T.B., Tomoda B.T., de Moraes M.A. (2020). Evaluation of Diclofenac Sodium Incorporation in Alginate Membranes as Potential Drug Release System. Materialia.

[35] Marangoni Júnior L., Jamróz E., Gonçalves S.d.Á., da Silva R.G., Alves R.M.V., Vieira R.P. (2022). Preparation and Characterization of Sodium Alginate Films with Propolis Extract and Nano-SiO_2_. Food Hydrocoll. Health.

[36] Sobczyk A.d.E., Luchese C.L., Faccin D.J.L., Tessaro I.C. (2021). Influence of Replacing Oregano Essential Oil by Ground Oregano Leaves on Chitosan/Alginate-Based Dressings Properties. Int. J. Biol. Macromol..

[37] Rhim J.W. (2004). Physical and Mechanical Properties of Water Resistant Sodium Alginate Films. LWT.

[38] Mahcene Z., Khelil A., Hasni S., Akman P.K., Bozkurt F., Birech K., Goudjil M.B., Tornuk F. (2020). Development and Characterization of Sodium Alginate Based Active Edible Films Incorporated with Essential Oils of Some Medicinal Plants. Int. J. Biol. Macromol..

[39] Siripatrawan U., Harte B.R. (2010). Physical Properties and Antioxidant Activity of an Active Film from Chitosan Incorporated with Green Tea Extract. Food Hydrocoll..

[40] Kaczmarek B. (2020). Improving Sodium Alginate Films Properties by Phenolic Acid Addition. Materials.

[41] Lamke L.-O., Nilsson G.E., Reithner H.L. (1977). The Evaporative Water Loss from Burns and the Water-Vapor Permeability of Grafts and Artificial Membranes Used in the Treatment of Burns. Burns.

[42] Sutar T., Bangde P., Dandekar P., Adivarekar R. (2021). Herbal Hemostatic Biopolymeric Dressings of Alginate/Pectin Coated with Croton Oblongifolius Extract. Carbohydr. Polym. Technol. Appl..

[43] Türkoğlu G.C., Sark A.M., Karavana S.Y. (2021). Development of Textile-Based Sodium Alginate and Chitosan Hydrogel Dressings. Int. J. Polym. Mater. Polym. Biomater..

[44] Chen J., Wu A., Yang M., Ge Y., Pristijono P., Li J., Xu B., Mi H. (2021). Characterization of Sodium Alginate-Based Films Incorporated with Thymol for Fresh-Cut Apple Packaging. Food Control.

[45] Cheng M., Wang J., Zhang R., Kong R., Lu W., Wang X. (2019). Characterization and Application of the Microencapsulated Carvacrol/Sodium Alginate Films as Food Packaging Materials. Int. J. Biol. Macromol..

[46] Abu Bakar A.J., Ghazali C.M.R., Mat Amin K.A. (2018). Sodium Alginate/Ageratum Conyzoides Extract Film for Wound Dressing Materials. IOP Conf. Ser. Mater. Sci. Eng..

[47] Rezvanian M., Mohd Amin M.C.I., Ng S.F. (2016). Development and Physicochemical Characterization of Alginate Composite Film Loaded with Simvastatin as a Potential Wound Dressing. Carbohydr. Polym..

[48] Avila L.B., Barreto E.R.C., Moraes C.C., Morais M.M., da Rosa G.S. (2022). Promising New Material for Food Packaging: An Active and Intelligent Carrageenan Film with Natural Jaboticaba Additive. Foods.

[49] Chi W., Cao L., Sun G., Meng F., Zhang C., Li J., Wang L. (2020). Developing a Highly PH-Sensitive ĸ-Carrageenan-Based Intelligent Film Incorporating Grape Skin Powder via a Cleaner Process. J. Clean. Prod..

[50] Hassan A., Niazi M.B.K., Hussain A., Farrukh S., Ahmad T. (2018). Development of Anti-Bacterial PVA/Starch Based Hydrogel Membrane for Wound Dressing. J. Polym. Environ..

[51] Abbasi A.R., Sohail M., Minhas M.U., Khaliq T., Kousar M., Khan S., Hussain Z., Munir A. (2020). Bioinspired Sodium Alginate Based Thermosensitive Hydrogel Membranes for Accelerated Wound Healing. Int. J. Biol. Macromol..

[52] Chaurasia V., Bajpai S.K. (2013). Moisture Uptake Behavior, Antibacterial Property, and Heat of Sorption of Nano Silver-Loaded Calcium Alginate Films. Int. J. Polym. Mater. Polym. Biomater..

[53] Salisu A., Musa Yar’ U., Sanagi M.M., Naim A.A., Juhanni K., Karim A. (2015). Graft Copolymerization of Methyl Methacrylate onto Alginate Using Benzoyl Peroxide Initiator. Res. J. Pharm. Biol. Chem. Sci..

[54] Wei J., Xu D., Zhang X., Yang J., Wang Q. (2018). Evaluation of Anthocyanins in Aronia Melanocarpa/BSA Binding by Spectroscopic Studies. AMB Express.

[55] Alzarea A.I., Alruwaili N.K., Ahmad M.M., Munir M.U., Butt A.M., Alrowaili Z.A., Shahari M.S.B., Almalki Z.S., Alqahtani S.S., Dolzhenko A.V. (2022). Development and Characterization of Gentamicin-Loaded Arabinoxylan-Sodium Alginate Films as Antibacterial Wound Dressing. Int. J. Mol. Sci..

[56] Dong Z., Wang Q., Du Y. (2006). Alginate/Gelatin Blend Films and Their Properties for Drug Controlled Release. J. Memb. Sci..

[57] Fata Moradali M., Donati I., Sims I.M., Ghods S., Rehm B.H.A. (2015). Alginate Polymerization and Modification Are Linked in *Pseudomonas aeruginosa*. mBio.

[58] Singleton V.L., Orthofer R., Lamuela-Ravent6s R.M. (1999). [14] Analysis of Total Phenols and Other Oxidation Substrates and Antioxidants by Means of Folin-Ciocalteu Reagent. Methods Enzymol..

[59] Brand-Williams W., Cuvelier M.E., Berset C. (1995). Use of a Free Radical Method. to Evaluate Antioxidant Activity. Lebensm. Wiss. Technol..

[60] (2021). tandard Test Methods for Water Vapor Transmission of Materials 1.

[61] Gontard N., Guilbert S., Cuq J.-L. (1992). Edible Wheat Gluten Films: Influence of the Main Process Variables on Film Properties Using Response Surface Methodology. Food Sci..

[62] (2010). Standard Test Method for Tensile Properties of Thin Plastic Sheeting.

[63] Liu C., Li P., Xu Y.J., Liu Y., Zhu P. (2023). Synergistic Effects of Iron Alginate on Improving the Fire Safety and Mechanical Properties of Epoxy Resin/Ammonium Polyphosphate Composites. Macromol. Mater. Eng..

